# Methicillin- and Inducible Clindamycin-Resistant *Staphylococcus aureus* among Patients with Wound Infection Attending Arba Minch Hospital, South Ethiopia

**DOI:** 10.1155/2019/2965490

**Published:** 2019-04-01

**Authors:** Mohammedaman Mama, Addis Aklilu, Kassahun Misgna, Molla Tadesse, Eyerusalem Alemayehu

**Affiliations:** ^1^Department of Medical Laboratory Sciences, Madda Walabu University Goba Referral Hospital, Bale-Goba, Ethiopia; ^2^Department of Medical Laboratory Sciences, Arba Minch University, Arba Minch, Ethiopia; ^3^Black Lion Specialized Hospital, Addis Ababa University, Addis Ababa, Ethiopia; ^4^Kebridehar Primary Hospital, Ethio-Somale, Ethiopia; ^5^Arba Minch Hospital, Arba Minch, Ethiopia

## Abstract

**Background:**

Wound infection is one of the most common hospital-acquired infections. Different bacteria cause infection, of which *Staphylococcus aureus* is one of the known bacteria in causing infection with increased drug-resistant isolates.

**Objective:**

To assess the prevalence and antimicrobial susceptibility pattern of methicillin and inducible clindamycin-resistant *Staphylococcus aureus* among patients with wound infections attending Arba Minch Hospital.

**Methods:**

A facility-based cross-sectional study was conducted from April to June 2017. A pretested questionnaire was used to collect demographic data and clinical characteristics. Wound swabs were cultured and identified by standard techniques. Antibiotic susceptibility tests were performed by the Kirby–Bauer disc diffusion method. Methicillin resistance was detected using the cefoxitin (30 *μ*g) antibiotic disc while inducible clindamycin resistance was detected by the D-zone test. The data were analyzed using Statistical Package for Social Science, version 20. *p* value <0.05 was considered statistically significant.

**Results:**

A total of 161 patients were enrolled and a majority of them were female (90, 50.9%). Among the collected samples, 79 (49.7%) were positive for *S. aureus*; of this, methicillin resistance accounted for 65 (82.3%). Out of 22 (27.8%) erythromycin-resistant isolates, 19 (24.1%) showed inducible clindamycin resistance. Methicillin-resistant *S. aureus* showed higher resistance against tetracycline (72.3%) followed by cotrimoxazole (43.1%) and 100% sensitivity to vancomycin. The overall prevalence of inducible clindamycin resistance among methicillin-resistant isolates was 16 (24.6%).

**Conclusion:**

The increasing prevalence of methicillin-resistant *S. aureus* and the coresistance against other therapeutic options like clindamycin is becoming an obstacle in the treatment of infections which need attention from concerned bodies.

## 1. Introduction

Wound infection is one of the known hospital-acquired infections responsible for significant human mortality and morbidity worldwide [1]. Wound infection results in sepsis, disfiguring, amputation, limb loss, long hospital stays, and higher costs [2, 3]. Infections of wounds can be caused by different microorganisms, like *Staphylococcus aureus* (*S. aureus*), *Pseudomonas aeruginosa* (*P. aeruginosa*), *Escherichia coli* (*E. coli*), and *Enterococcus* [4, 5]. *Staphylococcus aureus* is a Gram-positive bacterium which is a major causative agent of different skin infections such as surgical site infections, burns, and wounds [6, 7].

Methicillin-resistant *S. aureus* (MRSA) is a highly infectious strain of the ordinary *S. aureus* bacteria that is able to withstand the curative ability of commonly used antibiotics. Methicillin resistance of *S. aureus* is due to the acquisition of mecA gene that encodes for penicillin-binding protein 2a, which has low affinity to methicillin. Methicillin-resistant *S. aureus* is a serious concern not only because of resistant to methicillin but also because of developing resistant to other commonly used antibiotics in the hospitals that limited therapeutic options to few expensive drugs like vancomycin [7–9].

The increasing incidence of methicillin resistance among *Staphylococci* has led to renewed interest in the usage of macrolide-lincosamide-streptogramin B (MLSB) antibiotics to treat *S. aureus* infections, with clindamycin being the preferable agent due to its excellent pharmacokinetic properties [10, 11]. However, widespread use of MLSB antibiotics has led to an increase in the number of *Staphylococcal* strains acquiring resistance to MLSB antibiotics [12–14].

Methicillin-resistant *S. aureus* is found worldwide with an estimated colonization rate ranging from 11 to 40% in specific populations with more than 50% of these estimated to develop the infection [8, 15, 16]. Methicillin-resistant *S. aureus* infection kills more Americans each year than HIV/AIDS, Parkinson's disease, emphysema, and homicide combined in USA [15]. Despite the advances in modern medicine, wound infection still poses a risk of increased morbidity and mortality to patients. Even though many studies have analyzed the prevalence and antimicrobial susceptibility pattern of MRSA, there is still an increasing prevalence of MRSA [17]. Therefore, this study was aimed to assess the prevalence and antimicrobial susceptibility pattern of methicillin and induced clindamycin-resistant *S. aureus* among patients with wound infection attending Arba Minch Hospital.

## 2. Materials and Methods

### 2.1. Study Design, Area, and Period

A hospital-based cross-sectional study was conducted at Arba Minch Hospital, from April to June 2017. Arba Minch Hospital in Arba Minch town, Gamo Gofa Zone, is situated 505 km south of Addis Ababa at an elevation of 1285 meters above sea level. The hospital gives service for more than 100 thousand people in Arba Minch and surrounding woredas.

### 2.2. Sample Size Determination and Sampling Technique

The sample size was obtained using sample size determination formula for the estimation of the single population proportion. *p* value of 0.12 for MRSA was taken from the previous study [10] with 95% confidence interval (*z* = 1.96) and 5% marginal error (*d* = 0.05). The final sample size was 180 which includes 10% nonresponse rate. Systematic sampling technique was used to select patients with wound infection during the study period using *K*^th^ interval. The first patient was selected by the lottery method from the first three patients, and the rest of the study participants were selected in every 3 patients.

### 2.3. Data Collection, Sample Collection, and Transportation

A pretested structured questionnaire was used to collect sociodemographic data and clinical factors. Open wound swabs were aseptically obtained after the wound immediate surface exudates and contaminants were cleaned off with moistened sterile gauze and sterile normal saline solution. Dressed wounds were cleansed with sterile normal saline after removing the dressing. The specimen was collected on sterile cotton swab by rotating with sufficient pressure. The samples were transported to Medical Microbiology and Parasitology Laboratory of Arba Minch University within thirty minutes after collection using Amies transport media.

### 2.4. Inoculation and Identification

The collected samples were immediately processed for bacteriological analysis. Swabs collected were streaked on Mannitol salt agar by using a swab containing the sample on one-sixth of the media and then spread throughout the media by sterile inoculation loop. The plates were incubated at 37°C for 24–48 hours. Preliminary identification of bacteria was based on colony characteristics of the organisms like growth on Mannitol salt agar, gram reaction, and catalase and slide coagulase and test tube coagulase for slide coagulase-negative test results.

### 2.5. Detection of MRSA

Methicillin-resistant *S. aureus* was identified phenotypically based on its resistance to oxacillin (1 *μ*g) and cefoxitin (30 *μ*g) (Oxoid, Basingstoke, UK) by the disc diffusion method performed on modified Muller-Hinton agar (Oxoid, Basingstoke, UK). Based on the CLSI, 2016 guideline, the zone of inhibition is interpreted and grouped into methicillin-sensitive and methicillin-resistant *S. aureus* [18].

### 2.6. Detection of Inducible Clindamycin Resistance

A lawn culture of the isolates adjusted to 0.5 McFarland's turbidity was made on a Mueller-Hinton agar plate, and discs of clindamycin (2 *μ*g) and erythromycin (15 *μ*g) (Oxoid, Basingstoke, UK) were placed at a distance of 15 mm apart as per the Clinical Laboratory Standard Institute (CLSI, 2016) recommendations, along with routine antibiotic susceptibility testing. This interpretation was done only for erythromycin-resistant *S. aureus* strains. Induction test results were read at 16 to 18 h.

D phenotype (inducible MLSB) erythromycin (ERY)resistant (R), clindamycin (CLI) sensitive (S) (blunted, D-shaped clear zone around CLI disk proximal to the ERY disk); D^+^ phenotype (inducible MLSB) ERY R, CLI S (blunted, D-shaped zone around CLI disk proximal to the ERY disk and small colonies growing to CLI disk in otherwise clear zone); Neg phenotype (MSB) ERY R, CLI S (clear zone around CLI disk); HD phenotype (constitutive MLSB) ERY R, CLI R (two zones of growth appear around CLI disk: one zone is light, hazy growth extending from the CLI disk to the second zone where the growth is much heavier; the inner, hazy zone is blunted proximal to the ERY disk as in phenotype D; R phenotype (constitutive MSB): no hazy zone, growth up to CLI and ERY disks; S phenotype (no resistance) ERY R, CLI S (clear, susceptible zone diameters). All isolates showing positive induction test results (i.e., a blunted or “D-shaped” zone) and a subset of isolates with other induction test results were read again at 24 h [18].

### 2.7. Antimicrobial Susceptibility Testing

Antimicrobial susceptibility testing was performed by Kirby–Bauer disk diffusion technique according to the criteria set by CLSI (2016). Two to five pure colonies were transferred into a tube containing 5 ml nutrient broth and mixed gently until it forms a homogenous suspension. Then, turbidity of the suspension was adjusted to the optical density of McFarland 0.5 tubes in order to standardize the inoculum size. A sterile cotton swab was then dipped into the suspension. The swab was then used to distribute the bacteria suspension evenly over the entire surface of Mueller-Hinton agar. Antibiotics (Oxoid, Basingstoke, UK) which are found around the study area and recommended by CLSI for susceptibility test were erythromycin (15 *μ*g), ciprofloxacin (30 *μ*g), chloramphenicol (30 *μ*g), trimethoprim-sulfamethoxazole (cotrimoxazole) (1.25/23.75 *µ*g), amikacin (10 *μ*g), clindamycin (2 *μ*g), tetracycline (30 *μ*g), gentamicin (10 *μ*g), and vancomycin (E-test for MIC) (Oxoid, Basingstoke, UK). Then, the inoculated plates were left at room temperature to dry for 3–5 minutes. Using a sterile forceps, the antibiotic discs were placed on the inoculated plates and then incubated at 37°C for 18–24 hours. The diameter of the zone of inhibition around the disc was measured to the nearest millimeter using a ruler, and the isolates were classified as sensitive and resistant using CLSI standard [18].

### 2.8. Data Quality Control

Data quality was ensured from data collection up to final laboratory identification by following the prepared standard operating procedure (SOP). Five percent of the questionnaire was pretested prior to data collection in Arba Minch Health Center and modified accordingly. Data collection process was monitored on daily basis, and incompletely filled questionnaires were discarded. The performance of the prepared media was checked by inoculating control strain *S. aureus* ATCC 29213, which was obtained from Ethiopian Public Health Institute (EPHI). Culture media were prepared according to the manufacturer's instruction, and the sterility was checked by incubating 5% of prepared media at 37°C overnight and observing bacterial growth. Those batches of the media that show the growth was discarded and reprepared. The performance of antibiotic discs was checked by using *Enterococcus faccalis* ATCC 29122 and cotrimoxazole disc, and it should measure the inhibition zone greater than 19 mm.

### 2.9. Data Analysis

Data were collected, entered, cleaned, and analyzed using SPSS version 20 software according to the study objectives. The descriptive summaries were presented with text, tables, and figures. Binary logistic regression analysis was made to obtain odds ratio and confidence interval of statistical associated variables. All variables with *p* < 0.25 in the bivariate analysis were included in the final multivariate analysis. *p* value less than 0.05 was considered as statistically significant. Finally, the magnitude of association between different variables in relation to the outcome variable was measured by odds ratio with 95% confidence interval.

## 3. Results

### 3.1. Overall Sociodemographic and Clinical Characteristics of Study Participants

A total of 161 patients were enrolled in this study with a nonresponse rate of 11.6%. The majority of the study participants were female (90, 55.9%). According to age category, patients in the age range of 15–30 years account the most (80, 49.7%). The mean age of the study participants was 23.8 years (sd ± 11.24). The majority of study participants were urban dwellers (96, 59.6%), whereas 105 (65.2%) were literate. Study participants who developed wound infection after surgery were 53 (32.9%), and more than 90% of the study participants had the history of previous antibiotic usage for the last one year (Table 1).

### 3.2. Prevalence of MRSA among Sociodemographic and Clinical Characteristics

From 161 cultivated samples, a total of 79 (49.7%) isolated *S. aureus* isolates were isolated and screened for methicillin resistance as described in Section 2. Of this, methicillin-resistant *S. aureus* accounts 65 (82.27%) isolates, while the remaining isolates were methicillin-sensitive *Staphylococcus aureus* (MSSA). Majority of the MRSA isolates were recovered from male participants (35, 53.84%), urban dwellers (45, 69.23%), in the age range of 16–30 years (30, 46.15%). The participants who had a skin lesion and surgery showed a greater acquisition of MRSA (19, 29.23%) and (20, 30.54%), respectively (Tables 2 and 3).

### 3.3. Prevalence of MRSA and Associated Factors

As shown in Tables 2 and 3, different sociodemographic factors were assessed for possible association with MRSA infection among the study participants. The results of the study showed that 53.8% of male and 46.6% female participants were found to be infected with MRSA. The prevalence of infection with MRSA has an initial association with the sex of the respondents (*p*=0.041, COR: 0.514, 95% CI (0.271–0.975)). However, the association was not significant after adjusting for confounders using multivariate logistic regression (*p*=0.117). Study participants residing in urban area were found to have a high percentage (69.2%) of infection as compared to rural area. The association between residence and MRSA infection was statistically significant (*p*=0.042, COR: 0.504, 95% CI (0.260–0.976)). However, after adjusting for possible confounders by multivariate logistic regression, the prevalence of infection was not found to be statistically significantly different among urban and rural residents (*p*=0.172). Generally, there was no statistically significant association between the prevalence of MRSA and associated factors.

### 3.4. Antimicrobial Susceptibility Pattern

Identified isolates of *S. aureus* were tested against nine antibiotics as presented in Table 4. Both methicillin-resistant and susceptible *S. aureus* showed 100% susceptibility to amikacin and vancomycin. Furthermore, methicillin-sensitive *S. aureus* showed an additional 100% susceptibility to clindamycin and gentamycin. On the other hand, all isolates showed greater resistance against tetracycline (56, 70.9%), cotrimoxazole (31, 39.2%), and erythromycin (22, 27.8%). Specifically, MRSA strains showed the high resistance of tetracycline (72.3%), cotrimoxazole (43.1%), erythromycin (29.2%), and chloramphenicol (27.7%) and least resistance to clindamycin (3.1%).

### 3.5. Prevalence of Inducible Clindamycin Resistance

**Figure 1:**
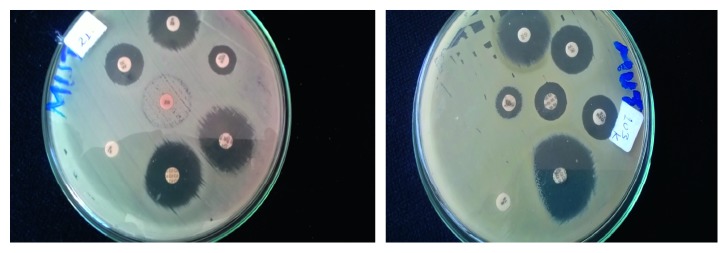
Disk diffusion technique showing D-test inducible clindamycin resistance from wound-infected patients attending Arba Minch Hospital, Arba Minch, South Ethiopia, April to June 2017.

From 79 *S. aureus* isolates tested for determination of inducible clindamycin resistance, 65 (82.3%) were MRSA and 14 (17.3%) isolates were MSSA (Table 3). Sensitivity to both erythromycin and clindamycin was significantly higher in MRSA compared to MSSA isolates. Resistance to methicillin, erythromycin, and clindamycin was observed in 65 (82.3%), 22 (27.8%), and 2 (2.53%) of the isolates, respectively. Inducible clindamycin resistance was determined in 19 (24.1%) isolates (D-test positive, Figure 1).

As shown in Table 5, inducible MSL_B_ phenotype predominated (24.6% MRSA; 21.4% MSSA) followed by cMLS_B_ (3.1% MRSA; 0% MSSA) and MS phenotypes (1 MRSA; 0 MSSA).

### 3.6. Prevalence of Multidrug-Resistant Isolates

**Figure 2:**
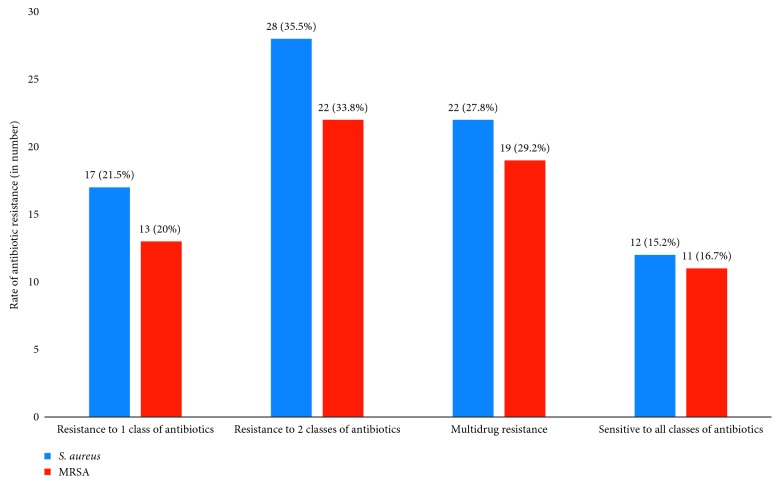
Antibiogram of *S. aureus* and MRSA isolated from patients with wound infection attending Arba Minch Hospital, Arba Minch, South Ethiopia, April to June 2017.

In this study, multidrug-resistant (MDR) status of *S. aureus* was tested against 9 classes of antibiotics. Accordingly, the overall rate of MDR (three and greater than three classes of antibiotics) of *S. aureus* isolates was 27.8% and 29.2% for MRSA. In addition to this, 20% of MRSA isolates showed resistance to one antibiotic class and 16.9% were sensitive to all checked antibiotic classes (Figure 2).

## 4. Discussion

Methicillin-resistant *S. aureus* has long been recognized as an important pathogen in human disease and is the most common cause of nosocomial infections [19]. The development of resistance against the therapeutic options for treatment of infection caused by MRSA is an emerging problem [1, 2, 7]. Therefore, this study was aimed to assess the prevalence and antimicrobial susceptibility pattern of methicillin-resistant and inducible clindamycin-resistant *S. aureus* among patients with wound infection.

This study showed that the recovery rate of MRSA was greater in male (35, 53.84%) patients with surgery (20, 30.54%) followed by patients with an infection after skin abrasion (19, 29.23%). Other studies performed in Jordan [20] and Uganda [21] reported the same result as in the present study. This may be attributed to the fact that men are mainly involved in occupations that most likely lead to trauma formation compared to women.

Out of 65 MRSA isolates, 30 (46.23%) were recovered from patients in the age range of 15–30 years. This is in line with a study conducted in Bangalore, India [14]. Patients who were hospitalized for more than 1 week harbored more MRSA isolates and those who took antibiotics previously within the last one year were found to be high and isolation of MRSA was also high. This is in agreement with the research performed in Cameroon [22] and India [14]. However, there was no statistically significant association between the prevalence of MRSA and associated factors in this study. This may be due to the smaller sample size included in our study.

Regarding associated factors assessed in the present study, wound infection was not associated with associated factors like use of antibiotics and previous wound infection known to predispose to infection. This finding is in agreement with the previous study performed in Greece [23] and Ethiopia [24]. Similarly, sociodemographic factors (age, sex, and educational status) did not show statistically significant association with wound infection which is in line with the study performed in Hawassa [25] and DebreMarkos [24].

In the present study, the overall prevalence of *S. aureus* was 79 (49.7%) which was in agreement with the study done in Hatay, Turkey [9], Kumasi, Ghana [26], and Yekatit 12 Hospital, Addis Ababa, Ethiopia [27]. Studies conducted in Nepal [28] and Kenya [29] show higher prevalence than the present study. Our result is higher when compared with researches performed in other parts of Ethiopia [24, 25] and Africa like Libya [30], Cameroon [22], and Tanzania [31]. These differences might be due to study design, period, and socioeconomic status of the population studied.

In this study, out of 79 isolated *S. aureus* isolates, 65 (82.28%) were MRSA isolates. It is comparable to studies performed in Southwest Ethiopia [32] and Nairobi, Kenya [29]. However, it is higher than the research report from Amhara, Ethiopia [24] Turkey [9], India [33–35], Nepal [28], Jordan [20], and Pakistan [36]. Furthermore, different studies in Africa too have depicted variations in the prevalence rates of MRSA in different countries [10, 22, 30, 31, 37]. This might be due to the variation in the population studied and the practice of antibiotics usage, sample size, sample type, and infection control practices.

Since the treatment of wound infection is on an empirical basis with first-line broad-spectrum antibiotics and the increase of drug resistance among pathogens causing wound infection especially *S. aureus* exists, continuously updated data on antimicrobial susceptibility patterns would be beneficial for the trend of empirical therapy. In this study, susceptibility of isolates was done on nine selected antibiotics by the disk diffusion technique which showed that MRSA tends to be resistant to a wider range of antibiotics.

In this study, MRSA isolates were showing higher resistance to tetracycline (72.3%), cotrimoxazole (43.1%), and erythromycin (29.2%). This was consistent with reports in Ethiopia [27] and elsewhere [7, 10]. The same isolate was highly sensitive to amikacin (100%), vancomycin (100%), clindamycin (96.9%), and gentamycin (94%) which is also in agreement with the research done in Tanzania [31] that reported 100% sensitivity to vancomycin and clindamycin, respectively. Remarkable susceptibility to vancomycin, amikacin, and gentamicin may be due to lesser use of these antibiotics as a result of their less availability and low cost.

In this study, vancomycin was 100% effective against both methicillin-resistant and -sensitive *S. aureus*. This was not in parallel with studies conducted in Addis Ababa, Ethiopia [27], DebreMarkos, Ethiopia [24], and Kumasi, Ghana [26] which reported the variable resistance of MRSA against vancomycin.

The present study revealed that out of 79 isolated *S. aureus* tested for inducible clindamycin resistance, 19 (24.1%) were positive (D^+^- and D-test positive). This is comparable with a study conducted in Mwanza, Tanzania (28.8%) [31]. But, it is higher than that of a study conducted in Nigeria (11.2%) [10] and Bangalore, India (9.15%) [14], however lower than a study conducted in India showing the prevalence of 47.12% and 41.3%, respectively [33, 35].

The overall prevalence of inducible clindamycin resistance among MRSA isolates were 16 (24.6%), whereas among MSSA, only 3 (21.4%) isolates showed inducible clindamycin resistance. This is supported by other studies performed in Andhra, South India (23, 28.04%) [34]. On the other hand, there was a higher prevalence of 61% [31], 87.8% [33], and 54.5% [35] of MRSA exhibiting inducible clindamycin resistance in Tanzania and India, respectively.

In this study, the remaining MRSA isolates showed another phenotype like no resistance phenotype (46, 70.8%), cMLS_B_ (2, 3.08%), and MS (D-negative) (1, 1.54%). Constitutive phenotype prevalence was in agreement with the study conducted by Mshana et al. in Tanzania [31] and higher than that of the report by Parasa et al. [34] and Vivek et al. [33] performed in India. In general, it may be risky to use clindamycin when erythromycin testing shows resistance or intermediate even though the bacteria are sensitive to clindamycin. For this reason, routine D-testing might help clinicians to retain confidence in using clindamycin when erythromycin resistance is observed [13].

In this study, the prevalence of MDR rate of *S. aureus* isolates was 27.8%. This prevalence was slightly lower than that of the study from Ethiopia reporting 34% [38] and 32.1% in Ghana [26] and more lower than that of the report from Jimma, Ethiopia (86.3%) [32]. The possible reasons for the prevalence differences may be attributed due to type of study population and study period and MDR definition disparity may also be probable reason.

## 5. Conclusion

The prevalence of MRSA in Arba Minch Hospital was found to be high. It is an alarming result which needs a due attention and intervention to control the spread of drug-resistant organisms. Amikacin and vancomycin were 100% effective drugs against both MRSA and MSSA isolates. However, high level of resistance was observed to tetracycline and cotrimoxazole among MRSA isolates. The incidence of inducible clindamycin resistance was also found to be too high. This may limit the therapeutic options and may lead to treatment failure. In this case, it may be very important to evaluate the susceptibility pattern of MRSA periodically.

## Figures and Tables

**Table 1:** Sociodemographic and clinical characteristics of patients with wound infection attending Arba Minch Hospital, Arba Minch, South Ethiopia, April to June 2017.

Variables	Characteristics	Frequency, *n* (%)
Sex	Male	71 (44.1)
	Female	90 (55.9)

Age	≤15	24 (14.9)
	15–30	80 (49.7)
	30–45	32 (19.9)
	45–60	22 (13.7)
	≥60	3 (1.9)

Residence	Urban	96 (59.6)
	Rural	65 (40.4)

Education	Illiterate	56 (34.8)
	Literate	105 (65.2)

Occupation	Student	42 (26.1)
	Housewife	42 (26.1)
	Labor	9 (5.6)
	Employee	31 (19.3)
	Private	21 (13)
	Farmer	8 (5)
	Jobless	8 (5)

Clinical diseases	Hypertension	17 (10.6)
	TB	8 (5)
	Diabetes	20 (12.4)
	HIV	16 (9.9)
	No chronic disease	100 (62.1)

Type of wound	Trauma	17 (10.6)
	Burn	22 (13.7)
	Surgical site	53 (32.9)
	Skin abrasion	48 (29.9)
	Others	21 (13)

Previous wound infection	Yes	97 (60.2)
	No	64 (39.8)

Previous antibiotic usage	Yes	148 (91.8)
	No	13 (8.1)

Hospital stay	1 day	36 (22.4)
	2–4 days	61 (37.9)
	5–6 days	19 (11.8)
	>week	45 (28)

**Table 2:** Multivariate analysis of MRSA and sociodemographic factors from wound infected patients attending Arba Minch Hospital, Arba Minch, South Ethiopia, April to June 2017.

Variable	Negative no. (%)	Positive no. (%)	Crude OR (95% CI)	*p* value	Adjusted OR (95% CI)	*p* value
Sex						
Male	36 (37.5)	35 (53.4)	1.00		1.00	
Female	60 (62.5)	30 (46.6)	0.514 (0.271–0.975)	0.041	0.366 (0.104–1.287)	0.117

Residence						
Urban	51 (53.1)	45 (69.2)	1.00		1.00	
Rural	45 (46.9)	20 (30.8)	0.504 (0.260–0.976)	0.042	0.442 (0.137–1.425)	0.172

Educational status						
Illiterate	38 (39.6)	18 (27.7)	0.585 (0.296–1.154)	0.122	5.997 (0.652–55.152)	0.114
Literate	58 (60.4)	47 (72.3)	1.00		1.00	

Occupation						
Student	19 (19.8)	23 (35.3)	1.00		1.00	
Housewife	34 (35.4)	8 (12.3)	0.194 (0.073–0.518)	0.001	0.062 (0.006–0.680)	0.203
Labor	5 (5.2)	4 (6.2)	0.661 (0.155–2.813)	0.575	0.557 (0.067–4.640)	0.589
Employee	16 (16.7)	15 (23.1)	0.774 (0.305–1.963)	0.590	0.802 (0.191–3.374)	0.764
Private	14 (14.6)	7 (10.8)	0.413 (0.139–1.231)	0.112	0.118 (0.016–0.848)	0.340
Farmer	5 (5.2)	3 (4.6)	0.496 (0.105–2.347)	0.376	0.301 (0.016–5.785)	0.426
Others	3 (3.1)	5 (7.7)	1.377 (0.291–6.519)	0.687	2.728 (0.056–133.888)	0.613

**Table 3:** Multivariate analysis of MRSA and clinical factors from wound-infected patients attending Arba Minch Hospital, Arba Minch, South Ethiopia, April to June 2017.

Variable	Negative no. (%)	Positive no. (%)	Crude OR (95% CI)	*p* value	Adjusted OR (95% CI)	*p* value
Previous diseases						
Hypertension	6 (6.3)	11 (17.0)	2.638 (0.904–7.704)	0.076	2.72 (0.482–15.363)	0.257
Tuberculosis	5 (5.2)	3 (4.6)	0.863 (0.195–3.815)	0.846	0.000	0.998
Diabetes	10 (10.4)	10 (15.4)	1.439 (0.549–3.769)	0.459	0.686 (0.116–4.057)	0.678
HIV/AIDS	16 (16.7)	0	0.000	0.998	0.000	0.998
No chronic diseases	59 (61.4)	41 (63.1)	1.00		1.00	

Type of wound						
Trauma	7 (7.3)	10 (15.4)	2 (0.650–6.151)	0.227	1.276 (0.095–17.211)	0.854
Burn	9 (9.4)	13 (20.0)	2.02 (0.725–5.639)	0.178	13.752 (0.104–1815.1)	0.293
Surgical site infection	34 (35.4)	19 (29.2)	0.782 (0.351–1.746)	0.549	0.320 (0.01–10.686)	0.525
Abrasion and skin tear	28 (29.2)	20 (30.8)	1.00		1.00	
Others	18 (18.7)	3 (4.6)	0.233 (0.060–0.900)	0.035	0.000	0.998

Cause of wound						
Burn	10 (10.4)	14 (21.5)	2.864 (1.098–7.467)	0.031	0.226 (0.002–29.914)	0.550
Surgery	35 (36.5)	19 (29.2)	1.110 (0.521–2.365)	0.786	0.140 (0.002–8.007)	0.341
Gun shot	2 (2.1)	4 (6.2)	4.091 (0.095–24.07)	0.119	0.402 (0.014–15.418)	0.666
Bite	0	3 (4.6)	0.000	0.999	0.000	0.999
Injury	45 (47.0)	22 (33.8)	1.00		1.00	
Others	4 (4.2)	3 (4.6)	1.534 (0.316–7.458)	0.596	0.000	0.998

Site of wound						
Head	24 (25.0)	8 (12.3)	0.4 (0.139–1.147)	0.088	0.845 (0.137–5.205)	0.856
Neck	5 (5.2)	5 (7.7)	1.2 (0.291–4.947)	0.801	21.405 (0.941–487.06)	0.06
Abdomen	10 (10.4)	12 (18.5)	1.44 (0.487–4.255)	0.509	11.159 (0.733–169.916)	0.083
Shoulder	5 (5.2)	4 (6.2)	0.96 (0.218–4.228)	0.957	1.540 (00.076–31.062	0.778
Buttock	2 (2.1)	3 (4.6)	1.8 (0.265–12.228)	0.548	10.84 (0.436–269.42)	0.146
Genitalia	2 (2.1)	4 (6.2)	2.4 (0.385–14.968)	0.349	75.202 (1.843–3068.17)	0.202
Hand	18 (18.7)	15 (23.1)	1.00		1.00	
Leg	28 (29.2)	14 (21.5)	0.6 (0.235–1.534)	0.286	0.441 (0.082–2.368)	0.340
Others	2 (2.1)	0	0.000	0.999	0.000	0.999

Hospital stay						
1 day	26 (27.1)	10 (15.4)	0.850 (0.343–2.109)	0.726	0.257 (0.054–1.217)	0.087
2–4 day	42 (43.7)	19 (29.2)	1.00		1.00	
5–6 day	5 (5.2)	14 (21.5)	6.189 (1.948–19.66)	0.002	5.896 (1.063–32.698)	0.420
>1 week	23 (24.0)	22 (33.8)	2.114 (0.953–4.692)	0.066	1.359 (0.376–4.918)	0.640

Previous wound infection						
Yes	47 (48.9)	50 (76.9)	1.00		1.00	
No	49 (51.1)	15 (23.1)	3.475 (1.722–7.013)	0.001	0.605 (0.173–2.121)	0.433

**Table 4:** Antimicrobial susceptibility pattern of MSSA and MRSA from wound-infected patients at Arba Minch Hospital, Arba Minch, South Ethiopia, April to June 2017.

Isolate		Antimicrobial agents, *n* (%)								
		VA	Cd	E	AK	TE	C	CIP	COT	GEN
MSSA (*n* = 14)	S	14 (100)	14 (100)	11 (78.6)	14/100	5 (35.7)	9 (64.3)	12 (85.7)	11 (78.6)	14 (100)
	R	0	0	3 (21.4)	0	9 (64.3)	5 (35.7)	2 (14.3)	3 (21.4)	0

MRSA (*n* = 65)	S	65 (100)	63 (96.9)	46 (70.8)	65/100	18 (27.7)	47 (72.3)	59 (90.8)	37 (59.9)	61 (93.9)
	R	0	2 (3.1)	19 (29.2)	0	47 (72.3)	18 (27.7).	6 (9.2)	28 (43.1)	4 (6.1)

Total (*n* = 79)	S	79 (100)	77 (97.5)	57 (72.2)	79/100	23 (29.1)	56 (70.9)	71 (89.9)	48 (60.8)	75 (94.9)
	R	0	2 (2.5)	22 (27.8)	0	56 (70.9)	23 (29.1)	8 (10.1)	31 (39.2)	4 (5.1)

*Key*: VA = vancomycin, Cd = clindamycin, E = erythromycin, AK = amikacin TE = tetracycline, KF = chloramphenicol, CIP = ciprofloxacin, COT =  cotrimoxazole, GEN = gentamycin.

**Table 5:** Susceptibility patterns of isolated *S. aureus* against erythromycin and clindamycin from wound-infected patients at Arba Minch Hospital, Arba Minch, South Ethiopia, April to June 2017.

Phenotype	E^s^ and C^s^ no resistance, *n* (%)	E^r^ and C^s^ (D zone negative) MS_B_, *n* (%)	E^r^ and C^s^ (D zone positive) iMLS_B_, *n* (%)	E^r^ and C^r^ cMLS_B_, *n* (%)
*S. aureus* (*n* = 79)	57 (60.85)	1	19 (24.1)	2
MRSA (*n* = 65)	46 (70.8)	1	16 (24.6)	2
MSSA (*n* = 14)	11 (78.6)	0	3	0

*Key*. E^s^: erythromycin sensitive, C^s^: clindamycin sensitive, E^r^: erythromycin resistant, C^r^: clindamycin-resistant, MS: macrolide streptogramin B, iMLS_B_: inducible macrolide lincosamidestreptogramin B phenotype, cMLS_B_: constitutive macrolide lincosamidestreptogramin B phenotype.

## Data Availability

The data used to support the findings of this study are available from the corresponding author upon request.
